# New clinically relevant, orthotopic mouse models of human chondrosarcoma with spontaneous metastasis

**DOI:** 10.1186/1475-2867-10-20

**Published:** 2010-06-28

**Authors:** Jonathan CM Clark, Toru Akiyama, Crispin R Dass, Peter FM Choong

**Affiliations:** 1 Department of Orthopaedics and University of Melbourne Department of Surgery, St Vincent's Health, Melbourne, Australia; 2 Sarcoma Service, Peter MacCallum Cancer Institute, Melbourne, Australia

## Abstract

**Background:**

Chondrosarcoma responds poorly to adjuvant therapy and new, clinically relevant animal models are required to test targeted therapy.

**Methods:**

Two human chondrosarcoma cell lines, JJ012 and FS090, were evaluated for proliferation, colony formation, invasion, angiogenesis and osteoclastogenesis. Cell lines were also investigated for VEGF, MMP-2, MMP-9, and RECK expression. JJ012 and FS090 were injected separately into the mouse tibia intramedullary canal or tibial periosteum. Animal limbs were measured, and x-rayed for evidence of tumour take and progression. Tibias and lungs were harvested to determine the presence of tumour and lung metastases.

**Results:**

JJ012 demonstrated significantly higher proliferative capacity, invasion, and colony formation in collagen I gel. JJ012 conditioned medium stimulated endothelial tube formation and osteoclastogenesis with a greater potency than FS090 conditioned medium, perhaps related to the effects of VEGF and MMP-9. In vivo, tumours formed in intratibial and periosteal groups injected with JJ012, however no mice injected with FS090 developed tumours. JJ012 periosteal tumours grew to 3 times the non-injected limb size by 7 weeks, whereas intratibial injected limbs required 10 weeks to achieve a similar tumour size. Sectioned tumour tissue demonstrated features of grade III chondrosarcoma. All JJ012 periosteal tumours (5/5) resulted in lung micro-metastases, while only 2/4 JJ012 intratibial tumours demonstrated metastases.

**Conclusions:**

The established JJ012 models replicate the site, morphology, and many behavioural characteristics of human chondrosarcoma. Local tumour invasion of bone and spontaneous lung metastasis offer valuable assessment tools to test the potential of novel agents for future chondrosarcoma therapy.

## Background

Chondrosarcoma is a common primary bone sarcoma, which is second only to osteosarcoma in incidence. It most often arises in the over 40 age group [1], and is associated with significant mortality and morbidity [2,3]. Although a significant proportion of chondrosarcomas are low-grade, these are well-known to recur locally, even 5 years or more following the initial tumour resection. This often requires further wide-margin surgery, with the potential for significant morbidity [4].

One of the key problems in chondrosarcoma, giving rise to poor patient outcomes, is the cancer's lack of response to both chemotherapy [5] and radiotherapy [6], leaving surgery as the only treatment option. Although the reasons for this are yet to be fully defined, it is thought that a variety of factors are contributory, including p-glycoprotein expression [7].

A more in-depth understanding of chondrosarcoma biology will hopefully lead to new molecular agents which can target the pathways responsible for chondrosarcoma progression. However, in order to achieve this goal, more appropriate animal models using human chondrosarcoma cells are required as a means to rigorously test candidate molecular agents.

There are already a considerable number of animal chondrosarcoma models available, which utilise both animal and human chondrosarcoma cell lines [8]. Most of the recently described models involve subcutaneous implantation of cells which lacks pathogenic relevance to the human disease. There is one recently described model implanting human chondrosarcoma in the mouse skull [9], and while this is orthotopic, the skull is an uncommon site for chondrosarcoma [10]. This model is also technically demanding which limits widespread use. Consequently, new orthotopic models are required. Our laboratory has previously developed an intratibial mouse model of human osteosarcoma using the non-transformed, SaOS-2 cell line [11]. This has led to successful evaluation of novel agents such as PEDF [12], the DNAzyme Dz13 [13], which downregulates c-jun, and also uPAR DNAzyme [14]. Given that an endosteal, appendicular location is most common for chondrosarcoma in human patients [2], a similar approach in the mouse would benefit research of similar agents in chondrosarcoma.

The aim of this study was to develop new orthotopic models of human chondrosarcoma in the nude mouse, replicating intermediate to high-grade chondrosarcoma or low-grade chondrosarcoma. These models could then serve as a platform for testing of candidate drug molecules as well as shed more light on chondrosarcoma progression.

## Methods

### Cell culture

JJ012 and FS090 cells were provided by J.A Block, (Rush University Medical Centre, Chicago, USA). Cells were cultured in DMEM (Invitrogen, Carlsbad, CA) supplemented with 10% fetal calf serum (FCS) and 1% antimicrobials, and were removed from 75 ml culture flasks by incubating in 2 ml of trypsin/EDTA with type I collagenase (1 mg/ml) (Sigma-Aldrich, Australia) at 37°C for 5 minutes. To compare cell morphology, JJ012 and FS090 cells were grown to 70% confluence on 6 well plates, fixed in methanol and then stained with hematoxylin and eosin. Chondrosarcoma cell-conditioned media was harvested after exposure to cells (JJ012 or FS090) at 80% confluence for 48 h under the conditions described. Media was used for the HMEC-1 tube formation and osteoclastogenesis assays described below. HMEC-1 cells (Centre for Disease Control, Atlanta, GA) were cultured in MCDB131 medium (Invitrogen, Carlsbad, CA) supplemented with 10 ng/ml of epidermal growth factor (Sigma-Aldrich, Australia), and 1 μg/ml hydrocortisone (Sigma-Aldrich, Australia). The mouse monocyte cell line, RAW 246.7 (donated by Prof T.J. Martin, St Vincent's Institute, Melbourne, Australia), was grown in α-MEM (Invitrogen, Carlsbad, CA) supplemented with 10% FCS and 1% antimicrobials.

### Proliferation assay

JJ012 or FS090 cells were seeded in 96 well plates, with 5000 cells/well and 3 repeats used for each cell line and time point. At day 2 and day 4, cells were fixed in methanol, stained with propidium iodide (1:10,000) and the central field of each well was visualised at ×40 magnification using a Nikon Eclipse TE2000-U microscope (Nikon, Australia), and photographed with a SPOT Advanced camera and software (Diagnostic Instruments Inc., Sterling Heights, MI). Cells fluorescing red under a Y-2E/C filter were counted using Image J software (National Institutes of Health, USA).

### Invasion assay

The invasion assay was based on Dass et al. [11] with minor modifications. Boyden chamber inserts with 8 μm filter membranes (BD Biosciences, Australia) were coated with a 50 μl volume of 25% Matrigel/75% Opti-MEM (Invitrogen, Carlsbad, CA) and incubated at 37°C for 30 min in companion plates. JJ012 and FS090 cells were trypsinised, centrifuged and washed in 5 ml of OptiMEM to remove excess serum, then further centrifuged and resuspended in low serum medium (LSM) containing 0.5% FCS. 5 × 10^4 ^cells in 0.2 ml LSM volume were added to the Boyden chambers in triplicate. 0.8 ml of α-MEM with 10% FCS was added to each well to create a concentration gradient. The plate was incubated for 24 h at 37°C in 5% CO_2 _and then invading cells were stained with Quick-Dip (Froline, Sydney, Australia). The central region of each membrane was photographed at × 200 magnification and cells were counted manually.

### Colony formation assay

This was based on a method by Luu et al [15]. Briefly, wells of a 24-well plate were coated with type I collagen (BD Biosciences, Australia) at a concentration of 1.69 mg/ml in 2× α-MEM, supplemented with 20% FCS and 0.75% NaHCO_3_. To each well, a top layer of collagen I gel, containing 2000 suspended JJ012 or FS090 was added. Colonies greater than 10 cells were counted at day 5.

### Tube formation assay

2.5 × 10^4 ^HMEC-1 cells were incubated in 100 μL of harvested cell-conditioned medium, either from JJ012 or FS090, in wells of a 96-well plate (4 repeats), coated with 100 μL of 100% Matrigel (BD Biosciences, Australia). Images were acquired of HMEC-1 tube-like structures at 8 h, and the total number of tube structures was manually counted for each well. Apart from using conditioned cell medium from chondrosarcoma cell lines, this assay was based on that described by Phung and Dass [16].

### Osteoclastogenesis assay

For conditioned media assays, RAW 246.7 cells were plated at a density of 1.3 × 10^4^/well in a 96 well plate with α-MEM containing 10% FCS on day 0. On day 1, media was changed to harvested cell-conditioned media (from either JJ012 or FS090) containing 100 ng/ml rhRANKL (R & D Systems, Minneapolis, MN) and 10% fetal calf serum. On day 4, cells were fixed with 25% citrate, 56% acetone and 3% formaldehyde for 30 s at 37°C, and then stained with 200 μl of TRAP stain for 1 h at 37°C according to the manufacturer's protocol (Sigma-Aldrich, Australia). Cells were then washed in tap water, and lightly counterstained in hematoxylin. The number of positively-staining osteoclast-like cells were counted manually. For co-culture assays, JJ012, or FS090 cells were plated at a density of 1.0 × 10^4^/well in 96 well plates on day 0, and then on day 1 RAW 246.7 cells were seeded at 1.3 × 10^4^/well in media supplemented with 100 ng/ml rhRANK. On day 4 cells were fixed and stained with TRAP as described above. Control wells, containing 1.3 × 10^4 ^RAW 246.7 cells/well in media supplemented with 100 ng/ml rhRANKL on day 1 were used for comparison of osteoclastogenic potential.

### Western blotting

Equal cell numbers of JJ012 and FS090 were grown to 90% confluence and then total cell protein was extracted from cells with RIPA buffer (150 nM NaCl, 50 nM Tris pH 8.0, 1 mM EDTA, 0.1% SDS, and 1% Triton X-100) and a complete mini protease inhibitor cocktail (Roche, Mannheim, Germany). Samples, combined with NuPAGE LDS sample buffer (Invitrogen, Australia), were separated on 4-12% Bis-Tris NuPAGE gels (Invitrogen, Australia) and transferred to PVDF membranes (Invitrogen, USA). Antibodies to human VEGF, GAPDH (Santa Cruz Biotechnology, Santa Cruz, CA), and also RECK (R & D Systems, Minneapolis, MN) were applied. Bands were visualized using secondary antibodies conjugated with HRP and a chemiluminescent kit (ECL detection reagent, Amersham Pharmacia Biotech Australia).

### Gelatin zymography

JJ012 and FS090 cells were grown to 80% confluence and then washed in PBS and immersed in serum-free medium for 24 h. Medium was then harvested and centrifuged at 400 *g *for 5 min. Equal quantities of protein confirmed on protein assay (Pierce Biotechnology, Rockford, IL) were loaded onto Novex 10% gelatin gels (Invitrogen, Australia). Recombinant human MMP-2 and MMP-9 (R & D Systems, Minneapolis, MN) were loaded as positive controls. Following electrophoresis, gels were incubated in renaturing and developing buffers (Invitrogen, Australia), stained with 0.1% Coomassie brilliant blue and destained in 7.5% acetic acid and 10% ethanol. Gel images were obtained with a Bio-rad Universal Hood II, and Quantity One software (Bio-rad, Australia).

### In vivo methods

Animal experimentation was approved by St Vincent's Hospital Animal Ethics Committee. Female, 5-week old Balb/c nude mice were anaesthetised with ketamine (100 mg/kg) and xylazine (10 mg/kg). 2 × 10^4 ^JJ012 or FS090 cells were suspended in 10 μL of 50% Matrigel/PBS, and injected into the intramedullary canal of the left tibia (n = 5 per study group) or into the periosteum of the anterior tibia (n = 5 per study group). Cells were checked for viability using trypan blue exclusion prior to injection. Tissue volume around the proximal tibia was measured at weekly intervals with calipers in the mediolateral and anteroposterior planes [17] and expressed as a ratio of the injected to the non-injected limb. Tibial radiographs were taken at 35 kV using a cabinet system (FaxitronCorp., McMinnville, OR). After fixing in 4% paraformaldehyde, and decalcifying in 0.4 M EDTA/PBS, limbs were processed and embedded in paraffin, sectioned at 5 μm, and stained with hematoxylin and eosin. Formalin-fixed, paraffin-embedded lungs were sectioned at 5 μm, and 40 sections from the middle third of each lung were evaluated for metastases. In two limbs containing periosteal JJ012 tumours, TRAP staining was performed to detect the presence of osteoclasts. Tissue sections were de-paraffinised, rehydrated in an ethanol series, stained with TRAP (Sigma-Aldrich, Australia) for 1 h at 37°C, rinsed in tap water, lightly counterstained in hematoxylin and then mounted in 50% glycerol.

### Statistical analyses

Statistical significance was determined for both *in vitro *and *in vivo *data using a one-tailed Student's *t*-test. P values < 0.05 were considered significant.

## Results

### Cell morphology

**Figure 1:**
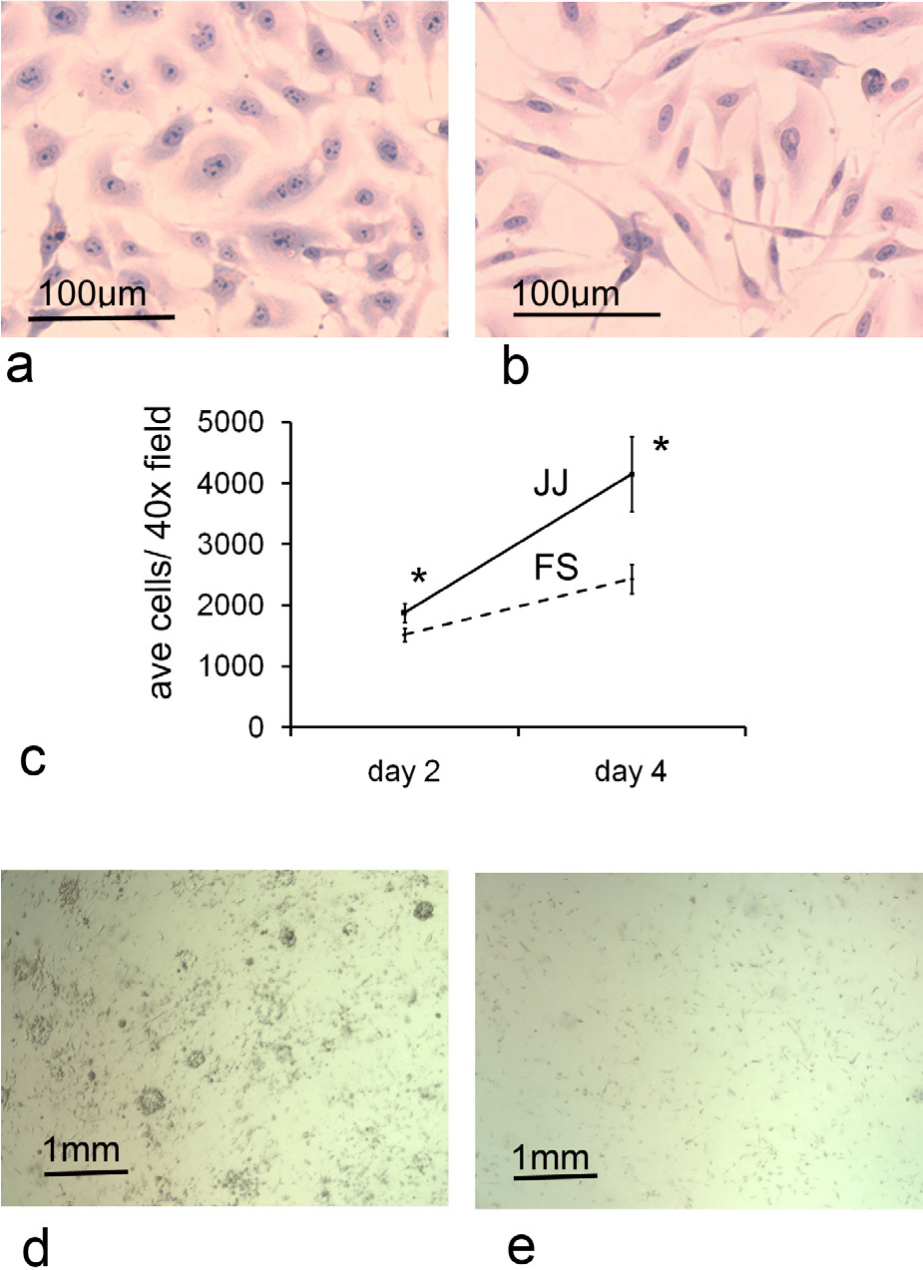
**Chondrosarcoma cell morphology and proliferation**. a) JJ012 demonstrated high pleomorphism and nuclear atypia with prominent nucleoli. b) FS090 had more distinct polarity and less nuclear atypia. c) JJ012 proliferated at a higher rate than FS090 (*p < 0.05). d) Prominent colony formation was noted for JJ012 suspended in collagen I gel. e) FS090 did not form colonies after 5 days.

JJ012 and FS090 cells stained with hematoxylin and eosin were found to demonstrate different morphological features. JJ012 were generally polyhedral in shape, with variable, ovoid nuclei and prominent nucleoli (Fig.1a). JJ012 cells lacked polarity in contrast with FS090, which were more spindle-shaped, and had less pleomorphic nuclei (Fig.1b).

### Proliferation assay

Overall, JJ012 demonstrated more aggressive behaviour *in vitro*. At day 2 and day 4 the average cell count for JJ012 was 1876 and 4155 respectively, versus 1521 and 2433 for FS090 (p = 0.017 at day 2, and p = 0.010 at day 4) (Fig.1c). From these average cell counts, the doubling time for JJ012 was calculated at 39.5 h versus 80 h for FS090.

### Colony formation assay

To further investigate proliferation in a matrix replicating bone, a collagen I colony formation assay was performed. JJ012 cells readily formed colonies by day 5 in collagen I gel (Fig.1d). By contrast FS090 showed no evidence of colony formation in collagen I (Fig.1e).

### Invasion assay

**Figure 2:**
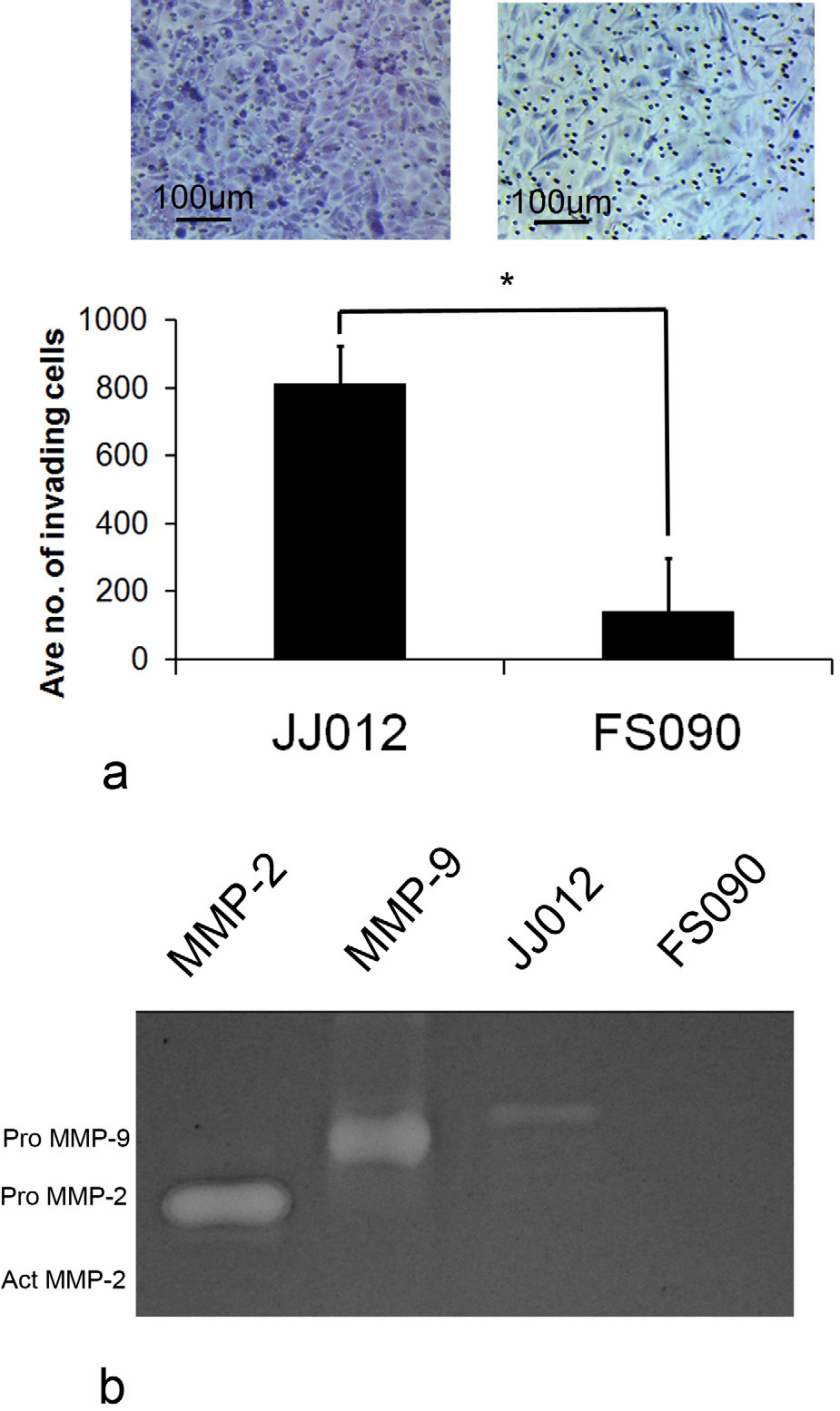
**Chondrosarcoma cell invasion**. a) A higher number of JJ012 cells invaded Matrigel compared with FS090 (* p < 0.05). b) Greater invasive capacity corresponded with the presence of pro MMP-9 in JJ012 conditioned media on gelatin zymography. This was in contrast with undetectable pro MMP-9 in FS090. No MMP-2 was detected for either cell line and no activated forms were present.

Invasion of matrix also predicts tumour progression, and at day 2 post seeding of 40,000 cells per Boyden chamber, an average number of 813 JJ012 cells were found to have invaded the Matrigel, versus 141 FS090 cells (p = 0.0005) (Fig.2a).

### Gelatin zymography

In an attempt to explain the differences in invasive capacity, cell-conditioned medium was analysed for gelatinase activity. Harvested medium from JJ012 was positive for pro MMP-9 but not pro or activated MMP-2, whereas FS090 medium yielded no detectable gelatinase bands (Fig.2b). MMP-2 and MMP-9 are important facilitators of cell invasion, and therefore the absence of these enzymes in FS090 is likely to explain the marked difference observed in the Matrigel invasion assay and also a lack of tumorigenesis.

### Angiogenesis assay and associated protein markers

**Figure 3:**
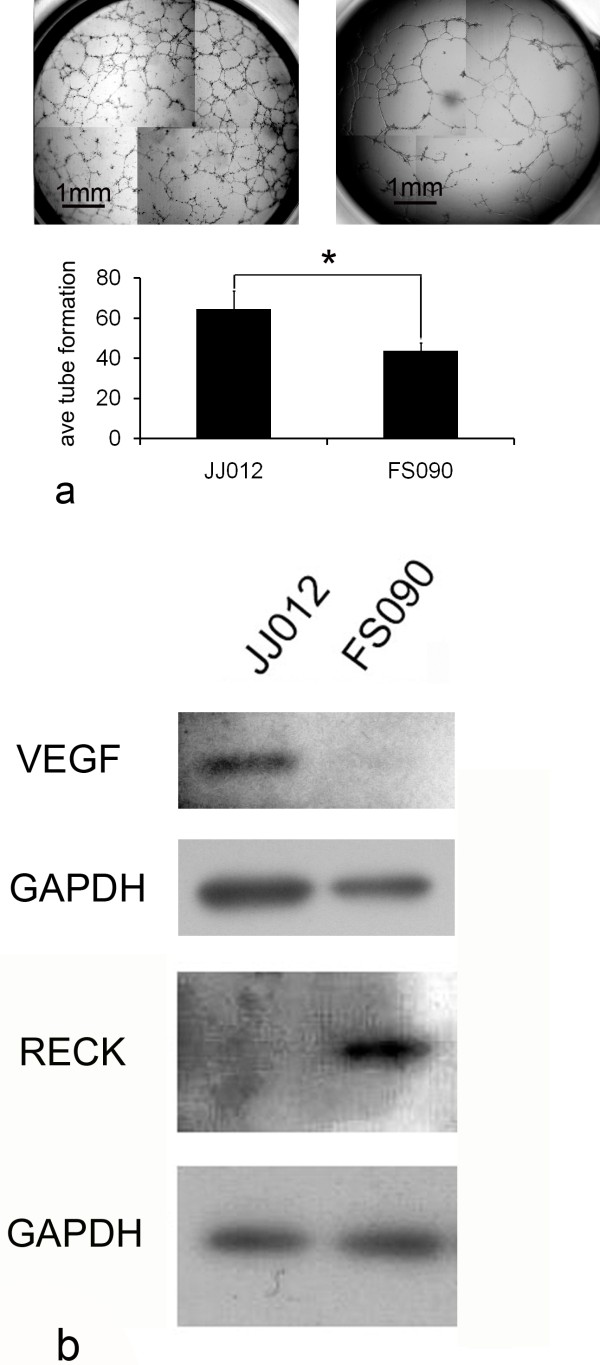
**Chondrosarcoma angiogenesis**. a) Conditioned media from JJ012 induced a higher number of tube formations compared with FS090 (* p < 0.05). b) Greater angiogenic capacity in JJ012 corresponded with the presence of VEGF on western blotting, in comparison with virtually undetectable VEGF in FS090. The anti-angiogenic protein RECK was prominently expressed by FS090, but not by JJ012.

Cell-conditioned medium from JJ012 stimulated HMEC-1 cells to form an average number of 64.5 tubes versus 43.8 for FS090 media (p = 0.007) (Fig.3a). The FS090 cultured medium was less angiogenic and this corresponded with virtually absent expression of VEGF on western blotting, in contrast with JJ012. FS090, on the other hand, produced significantly higher levels of RECK protein compared with JJ012 (Fig.3b), and this protein is known to inhibit MMP-2, MMP-9 [18], and possibly also VEGF [19], which may explain the absence of these target proteins in FS090.

### Orthotopic tumour growth and progression

**Figure 4:**
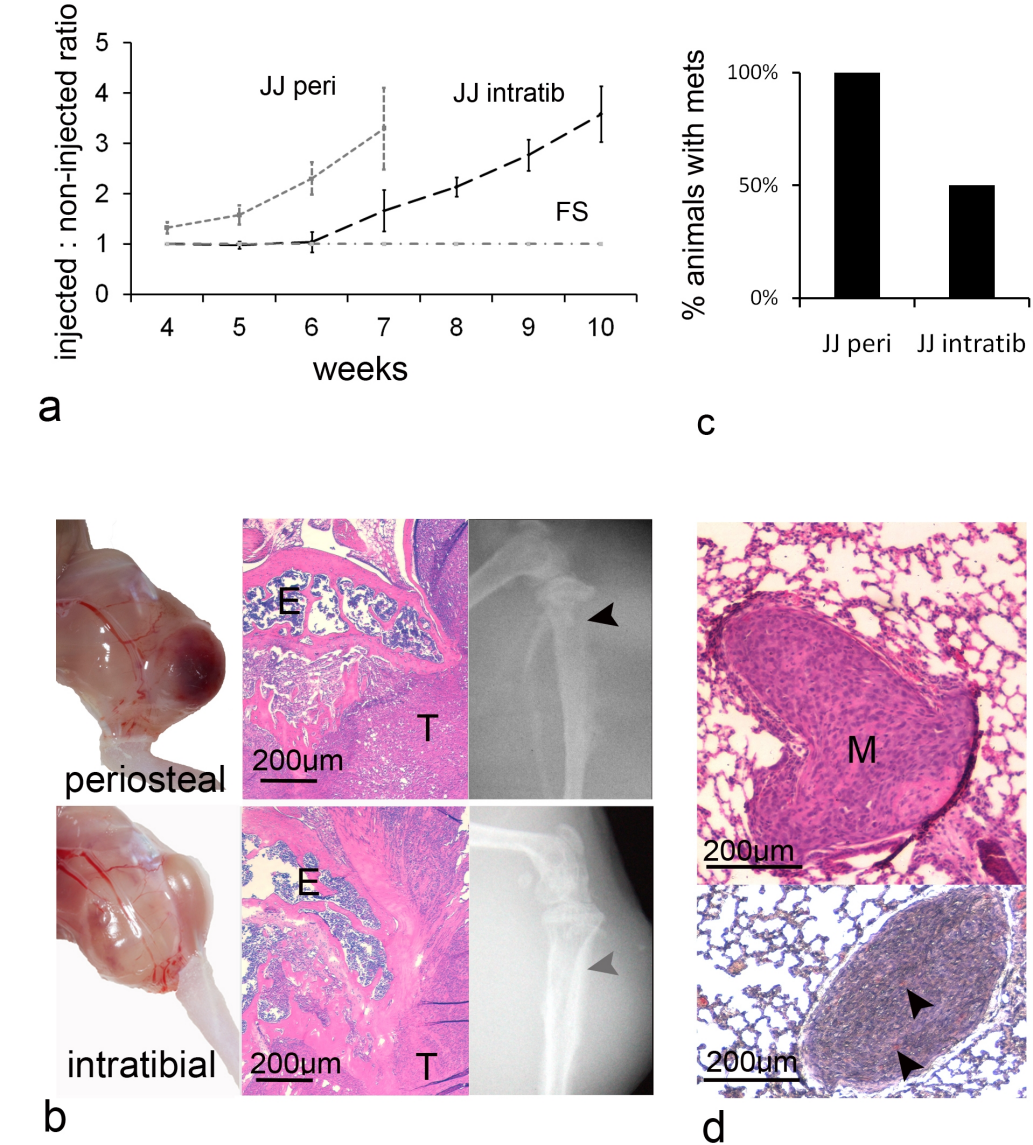
**In vivo growth and progression of chondrosarcoma cell lines**. a) This graph plots the injected to non-injected limb volume ratios for the study groups at each time point. Periosteal injected JJ012 developed into tumours most rapidly, followed 2-3 weeks later by intratibial injected JJ012. FS090 did not form tumours in either implantation site. b) Periosteal tumours expanded one area of the limb while intratibial tumours grew in a circumferential manner. On histology both tumours (T) invaded cortex, but leaving the epiphysis (E) largely intact. On radiographs, lytic regions (black arrow), and sclerotic (grey arrow) were noted in both models. c) 5/5 animals injected with periosteal JJ012 demonstrated lung metastases, in contrast with 2/4 animals in the intratibial group. d) Lung metastases (M) were lobular in appearance, and were positive on Alizarin red staining (arrows), indicating calcification.

JJ012 cells implanted within the periosteum developed into tumours by 4 weeks, while intratibial implanted JJ012 cells developed tumours between 6-7 weeks (Fig.4a). The average volume of JJ012 periosteal tumours was 174.2 mm^3 ^just prior to euthanasia at 7 weeks. Intratibial JJ012 tumours had an average volume of 198.5 mm^3 ^just prior to euthanasia at 10 weeks. By contrast, FS090 implanted either within periosteum or within the intramedullary canal did not form any tumours, even at 3 months (Fig.4a). Periosteal JJ012 tumours tended to expand into the soft tissues in one part of the limb, while intratibial tumours grew in a more circumferential manner (Fig.4b). At dissection, new vessels arising from the femoral artery and supplying the tumours were noted, and corresponding with the in vitro angiogenesis activity noted for JJ012. On sectioning of the proximal tibias, tumour tissue invaded the cortex and medulla of the metaphysis, while the epiphysis was largely preserved, consistent with the human disease (Fig.4b). Radiolucencies and opacities consistent with bony erosions and periosteal reactions respectively were found on x-ray (Fig.4b). All JJ012 periosteal tumours (5/5 animals) resulted in spontaneous lung metastases by 7 weeks while JJ012 intratibial tumours gave rise to lung metastases in 50% of animals (2/4) (Fig.4c). One of the original 5 animals in this latter group had been euthanised and excluded from the study due to systemic infection at week 6. Metastases were only visualised on microscopy (rather than macroscopically), and stained positive with Alizarin red, indicating calcification, which is also characteristic of human chondrosarcoma metastases (Fig.4d).

### Tumour histology and influence on osteoclasts

**Figure 5:**
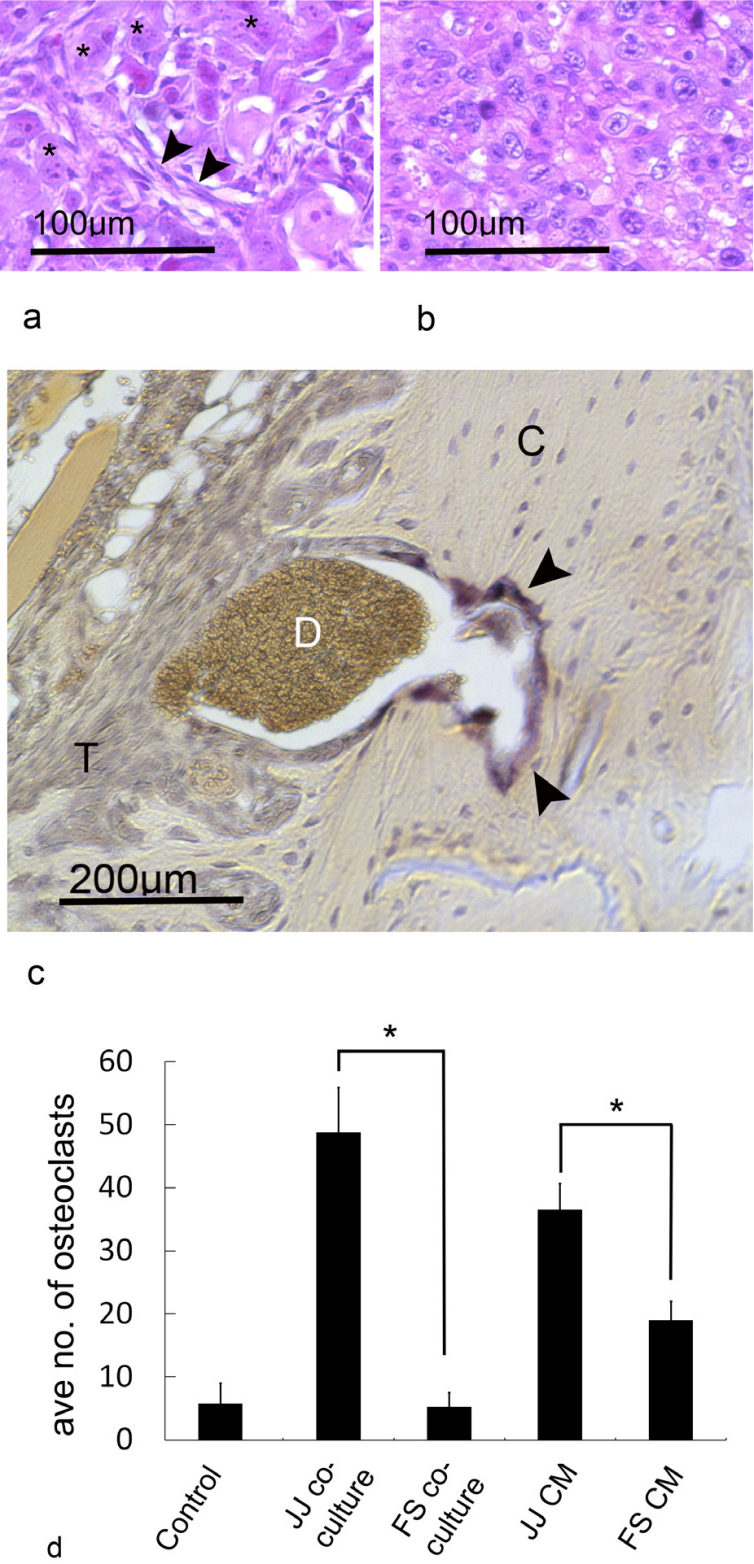
**Tumour histology and osteoclast involvement**. a) Pleomorphic, ovoid cells (*) with an additional spindle cell population (arrows) were noted for JJ012 tumours. b) Dedifferentiated chondrosarcoma from a human de novo tumour demonstrated some similar ovoid cells without a spindle cell component. c) In periosteal and intratibial tumours, TRAP staining (arrows) revealed prominent osteoclasts at the interface between tumour (T) and bone cortex (C), and this was associated with bone matrix erosion and a mass of dead cellular material (D). d) An osteoclastogenesis assay demonstrated higher osteoclasts formed by JJ012 media (CM) compared with FS090 media (CM) (* p < 0.05), and the same relationship for chondrosarcoma and mouse monocyte (RAW 246.7) co-culture (* p < 0.05).

JJ012 histology (either intratibial or periosteal) demonstrated an undifferentiated, high-grade appearance (Fig.5a) with pleomorphism and frequent nucleoli similar to a patient biopsy sample of dedifferentiated chondrosarcoma (Fig.5b). The main difference noted in the animal model was the addition of a spindle cell population of cells. TRAP staining of the JJ012 tumour tissue revealed TRAP-positive, osteoclast-like cells at the bone-tumour interface, which were associated with erosions of the cortex (Fig.5c). There were none of these cells in the cortex when tumour was not adjacent. These TRAP-positive cells were thought to be osteoclasts activated by the adjacent tumour.

### Influence of chondrosarcoma cell lines on osteoclasts

An osteoclastogenesis assay was then performed to quantify the influence of chondrosarcoma cells over osteoclast formation. Culture medium conditioned by either JJ012 or FS090 cells for 48 h was harvested and incubated with the osteoclast precursor cell line RAW 264.7. JJ012 media induced a significantly higher number of osteoclast-like cells (average of 37) in comparison with FS090 which resulted in an average of only 19 cells (p = 0.00035) (Fig.5d). A similar pattern was noted when the chondrosarcoma cell lines were co-cultured with RAW 264.7, and in this case JJ012 cells stimulated an average of 49 osteoclasts compared to 5 for FS090 (p = 0.00027). Control medium with RANKL stimulated an average of 6 osteoclasts. These results indicated that cell interactions and secreted humoral factors, by JJ012 in particular, were capable of significantly increasing the number of osteoclasts formed, which might aid bone resorption and tumour progression in vivo.

## Discussion

To our knowledge, these described models are the first clinically relevant orthotopic animal models of human chondrosarcoma. This is because they utilise a non-transformed human chondrosarcoma cell line, implanted into the native medullary or periosteal environment and consistently give rise to chondrosarcomas resulting in spontaneous lung metastases. Thus, the usual sequence of human chondrosarcoma progression is replicated, through proliferation, angiogenesis, invasion of cortical bone and soft tissues, and blood borne metastasis to the lung.

Of the seven most recently described chondrosarcoma xenograft models, involving human chondrosarcoma cell lines, only one was implanted into bone [9], albeit the cranium. The majority of chondrosarcoma animal models are subcutaneous in the back or flank and none of these human cell line models describe metastasis [8]. This could relate to a lack of metastases arising from these subcutaneous sites, or else due to the implanted cells being relatively indolent. The latter explanation is quite possible given that at least two of these models demonstrated features of intermediate grade histology with obvious chondroid matrix production [20,21].

Of the nine published allograft models using animal-derived chondrosarcoma, which are now infrequently employed, only two involve intramedullary tumour growth [22,23], and of these, only one model specifies lung metastases, occurring in 1/24 rats implanted [22]. Tumours in this aforementioned study were also described as well-differentiated, which is perhaps why metastasis occurred in only one animal.

Transgenic chondrosarcoma models have also been utilised with some success. Ho and colleagues have recently described a mouse model of transgenic enchondromatosis expressing Gli2 and further modified, via heterozygous knockout of p53, to produce low grade chondrosarcomas in 50% of animals [24]. Likwise, c-fos expression has been used to induce chondrosarcomas in around half of animals tested, with a mean latency of 9.5 months [25]. While these transgenic models can mimic the pathogenesis of human chondrosarcoma to some extent, their relatively inconsistent tumour growth reduces their power to test novel agents at this stage.

In relation to tumorigenic potential, significant differences in cell behaviour were identified between the two cell lines, JJ012 and FS090, examined by this study. Although both were derived from intermediate grade chondrosarcoma, JJ012 has less defined cytoplasmic polarity in contrast to the more spindle-shaped FS090, and possesses larger nuclei with frequent nucleoli. The original article concerning these cell lines focused on flow cytometry analysis and identified JJ012 as being hypoploid and the majority of FS090 being diploid [26]. Mankin and colleagues [27] demonstrated a strong association between ploidy and disease stage in musculoskeletal tumours, with the majority of benign tumours diploid, and the majority of stage III tumours aneuoploid. As previously noted, JJ012 is reported to have a consistent chromosome 9 monosomy, and this is significant given that a number of important tumour suppressor genes occupy this locus, including INK4A/ARF [28], PTCH1 [29], and the more recently described RECK gene which is downregulated in many common cancers [30,31].

A more recent study has attempted to define JJ012 and FS090 on the basis of differentiation, and the pattern of collagen markers suggests FS090 is the more differentiated cell line [32]. However, other comparisons of cell behaviour, including the influence on angiogenesis and osteoclastogenesis have not previously been performed for either cell line to our knowledge. This characterisation, in the present study, has provided a better understanding of each cell line's properties and explains the dramatic difference in tumorigenesis.

An increased cell proliferation rate for JJ012 corresponded with its more dedifferentiated morphology and prominent nucleoli. This, combined with the high propensity to form colonies in collagen I matrix (a measure of anchorage-independent growth), indicated strong potential for tumorigenesis within a bone environment [15]. Prominent anchorage-independent growth in JJ012 is likely related to its expression of anti-apoptosis proteins Bcl-2, Bcl-xL and XIAP [33]. On the other hand, the complete lack of colony formation in FS090 correlates well with its lack of tumorigenicity.

Vascular supply is also a major factor in tumorigenesis [34]. Studies have demonstrated that microvascular density increases with chondrosarcoma grade [35,36], and new anti-angiogenic agents are being used successfully in the experimental animal to reduce vessel development and also chondrosarcoma progression [9]. Therefore, we aimed to determine the influence of angiogenic-related factors produced by JJ012 and FS090 to explain differences observed in tumorigenesis. VEGF is well-known to stimulate migration of endothelial cells, and is a key protein in chondrosarcoma angiogenesis and its expression correlates with higher grade chondrosarcoma [35]. When medium from JJ012 or FS090 cells was used to suspend HMEC-1 with underlying Matrigel, tube formation was significantly lower in number for the FS090 medium-treated wells. This corresponded with virtually undetectable VEGF protein levels when western blotting was performed for FS090, while JJ012 was VEGF-positive. Lin et al [37] examined VEGF gene expression in both these cell lines and actually found higher VEGF gene expression in FS090, although when exposed to 24 hours of hypoxia, JJ012 produced a greater increase in VEGF gene expression than FS090. Furthermore, the level of HIF-1α in JJ012 was somewhat greater than for FS090 under hypoxic conditions [37]. Hypoxic conditions are more likely in JJ012 cell culture given the higher proliferation rate and closely packed nature which the cells display on culture plates. This may translate to more expressed VEGF protein demonstrated by this study.

One tumour suppressor with anti-angiogenic activity, via inhibition of gelatinases [18] and possibly also via inhibition of VEGF [19], is reversion-inducing, cysteine-rich protein with kazal motifs (RECK) [18,38]. This protein could only be detected in FS090 on western blotting (perhaps related to chromosome 9 preservation), and RECK was associated with absent MMP-9, which fits RECK's role as an MMP-9 inhibitor [38]. By contrast, JJ012, being a more aggressive cell line and having a known chromosome 9 monosomy, did not produce RECK, potentially leaving MMP-9 expression unchecked. These differences would further increase the tumorigenic potential of JJ012.

While *in vitro *studies demonstrated higher potential for tumour formation in the JJ012 cell line versus FS090, we decided to pursue investigation of both cell lines *in vivo *because of the value in having models with contrasting tumour grades. Both inoculation approaches, periosteal and intratibial/intramedullary, were used because chondrosarcoma arises in both sites clinically, although an intramedullary location is the most common. Two different approaches were hoped to maximise success of tumour growth and allow a comparison of growth patterns and progression according to anatomical location.

No tumour formation was observed for limbs injected with FS090. Although this was consistent with *in vitro *findings, it was surprising that FS090 did not grow even in the periosteal location, which provided a very conducive growth environment for JJ012. The FS090 cell line is certainly not as aggressive as JJ012, but it does possess an ability to invade Matrigel and stimulate angiogenesis, which are key elements of tumorigenesis. A lack of tumour growth in the nude mouse may relate to the combination of a differentiated morphology, a preserved chromosomal compliment, undetectable gelatinases and VEGF, and reduced osteoclastogenesis.

The reason for differences in osteoclastogenesis between the two cell lines is not fully defined but the presence of VEGF and MMP-9 in JJ012, and the lack of these proteins in FS090 provide a starting point. MMP-9 is known to play a prominent role in osteoclast recruitment [39], and also migration [40]. Also, because RECK is an inhibitor of MMP-9, the absence of RECK in JJ012 may contribute to a higher potential for MMP-9 mediated osteoclast effects. VEGF is important to osteoclast function by increasing osteoclast cell survival and bone resorption capacity [41], and can act as a substitute for M-CSF in stimulating osteoclastogenesis [42].

In regard to JJ012 tumorigenesis, the more rapid growth and progression of the periosteal tumour site is likely to relate to the developing tumour being unconfined by rigid bone, and perhaps a more direct vascular supply from the adjacent femoral vessels compared with intratibial implantation. Periosteum is known to have a very dense capillary network, even more so than skin and skeletal muscle, and with relatively large diameter capillaries [43]. This network contributes 80% of the arterial blood supply to the bone cortex as observed in guinea pig long bones [44]. On the other hand, the endosteal portion of the metaphysis (site of JJ012 intratibial injection) receives its arterial supply predominantly from one nutrient artery, which then divides within the bone, and from metaphyseal end-arteries which usually do not anastomose [45]. Sparing of the growth plate was noted for both implantation sites, and this anatomical structure has been shown to be a barrier to sarcoma invasion due to the presence of anti-angiogenic factors like PEDF [46].

Despite more rapid periosteal growth experimentally, the clinical comparison of periosteal and intramedullary chondrosarcoma is quite the opposite. Clinically, periosteal chondrosarcoma is a more indolent neoplasm, of low histological grade, low rate of lung metastases [47], and has an overall 5 year survival of 83% [48]. The discrepancy with our more aggressive model of periosteal chondrosarcoma clearly lies in the use of an aggressive/less-differentiated cell line (JJ012). In the more rare instances where clinical periosteal chondrosarcomas have demonstrated higher grade histology, the progression of these tumours has been suitably more aggressive with 50% of intermediate grade tumours resulting in metastases versus 6% of low grade tumours [48].

Although low grade orthotopic chondrosarcoma models should be sought, given the greater prevalence of low grade disease, it is equally advantageous to investigate novel agents in intermediate to high grade chondrosarcoma because this spectrum of disease has the most impact on patient survival, while still comprising over a third of all cases [49]. As the periosteal implantation more consistently produces lung metastases it should provide a suitable means to assess novel agents predicted to inhibit aspects of the metastatic cascade. For pharmaceutical drug R+D, tumour models that grow within 2-3 months, require non-exacting skills for model establishment, incorporate both primary and secondary tumours, and are viable/palpable non-invasively, present as the best to use. The JJ012 periosteal model fits these criteria. The intratibial model, on the other hand, may facilitate characterisation of interactions between chondrosarcoma and bone, and the influence of molecular agents on local tumour progression.

## Conclusion

The intratibial and periosteal models of chondrosarcoma described provide a useful representation of high grade chondrosarcoma in orthotopic sites. These models provide an assessment platform for future candidate therapies in chondrosarcoma. Furthermore, the models will facilitate more in-depth investigation into the biology of chondrosarcoma, particularly in terms of its interaction with bone.

## Competing interests

The authors declare that they have no competing interests.

## Authors' contributions

JCMC prepared the manuscript and performed the in vitro and in vivo studies, apart from the osteoclastogenesis assay and TRAP staining, which were performed by TA. CRD and PFMC supervised the overall study and assisted in the manuscript preparation. All authors have read and approved the final manuscript.
